# Supplementary material for: Protein/Fiber Index Modulates Uremic Toxin Concentrations in Hemodialysis Patients
Source: Toxins (Basel). 2022 Aug 27;14(9):589. doi: 10.3390/toxins14090589 (PMC9502511; doi:10.3390/toxins14090589)
Supplement: Supplementary file 1 [file toxins-14-00589-s001.zip › toxins-1827257-supplementary.pdf]

toxins

MDPI

Article

Protein/Fiber Index Modulates Uremic Toxin Concentrations in Hemodialysis Patients

Manon Ebersolt, Tacy Santana Machado, Cecilia Mallmann, Nathalie Mc-Kay, Laetitia Dou, Dammar Bouchouareb, Philippe Brunet, Stéphane Burtey and Marion Sallée

Table S1. Correlation between indoxyl sulfate and p-cresyl sulfate and dietary components in total population (n =58).

	Indoxyl sulfate		p-cresyl sulfate	
	test	*p*	test	*p*
Diuresis (non anuric-anuric)	*t* = −4.50	<0.0001*	*t* = 0.65	0.51
Gender (female-male)	*t* = −1.57	0.12	*t* = -0.88	0.38
Diabetes (yes-no)	*t* = −1.23	0.22	*t* = 1.64	0.11
Age (years)	r = -0.28	0.03*	r = 0,35	0.01*
Weigt (kg)	r = 0.16	0.23	r = 0.09	0.51
Dialysis vintage	r = 0.28	0.03*	r = -0.13	0.31
Energy (kcal/d)	r = -0.07	0.59	r = -0.21	0.12
Energy (kcal/kg/d)	r = 0.19	0.14	r = _0.21	0.10
Protein (g/d)	r = -0.12	0.37	r = 0.06	0.63
Protein (g/kg/d)	r = -0.28	0.03*	r = 0.01	0.97
Animal protein (g/kg/d)	r = -0.23	0.08	r = 0.15	0.27
Vegetal protein (g/kg/d)	r = -0.22	0.10	r = -0.22	0.10
Fiber (g/kg/d)	r = -0.25	0.06	r = -0.17	0.20
Fiber (g/d)	r = -0.10	0.45	r = -0.14	0.29
Protein / fiber index	r = 0.09	0.51	r = 0.18	0.18
Animal protein / fiber index	r = 0.07	0.60	r = 0.21	0.11
Vegetal protein / fiber index	r = -0.06	0.64	r = 0.03	0.8

Protein/fiber index is the ratio of total protein to total fiber. A data square root are transformed prior to spearman's correlation for continuous variable and student test for categorical variable, * $p < 0.05$.
